# Meat consumption and mortality - results from the European Prospective Investigation into Cancer and Nutrition

**DOI:** 10.1186/1741-7015-11-63

**Published:** 2013-03-07

**Authors:** Sabine Rohrmann, Kim Overvad, H Bas Bueno-de-Mesquita, Marianne U Jakobsen, Rikke Egeberg, Anne Tjønneland, Laura Nailler, Marie-Christine Boutron-Ruault, Françoise Clavel-Chapelon, Vittorio Krogh, Domenico Palli, Salvatore Panico, Rosario Tumino, Fulvio Ricceri, Manuela M Bergmann, Heiner Boeing, Kuanrong Li, Rudolf Kaaks, Kay-Tee Khaw, Nicholas J Wareham, Francesca L Crowe, Timothy J Key, Androniki Naska, Antonia Trichopoulou, Dimitirios Trichopoulos, Max Leenders, Petra HM Peeters, Dagrun Engeset, Christine L Parr, Guri Skeie, Paula Jakszyn, María-José Sánchez, José M Huerta, M Luisa Redondo, Aurelio Barricarte, Pilar Amiano, Isabel Drake, Emily Sonestedt, Göran Hallmans, Ingegerd Johansson, Veronika Fedirko, Isabelle Romieux, Pietro Ferrari, Teresa Norat, Anne C Vergnaud, Elio Riboli, and Jakob Linseisen

**Affiliations:** 1 Division of Cancer Epidemiology and Prevention, Institute of Social and Preventive Medicine, University of Zurich, 8001 Zurich, Switzerland; 2 Division of Cancer Epidemiology, Deutsches Krebsforschungszentrum, 69221 Heidelberg, Germany; 3 Section of Epidemiology, Department of Public Health, Aarhus University, 8000 Aarhus, Denmark; 4 National Institute for Public Health and the Environment (RIVM), 3720 Bilthoven, The Netherlands; 5 Department of Gastroenterology and Hepatology, University Medical Centre, 3508 Utrecht, The Netherlands; 6 Danish Cancer Society Research Center, 2100 Copenhagen, Denmark; 7 Inserm, Centre for Research in Epidemiology and Population Health, U1018, Institut Gustave Roussy, 94805 Villejuif, France; 8 Paris South University, UMRS 1018, 94805 Villejuif, France; 9 Nutritional Epidemiology Unit, Fondazione IRCCS Istituto Nazionale Tumori, 20133 Milan, Italy; 10 Molecular and Nutritional Epidemiology Unit, Cancer Research and Prevention Institute (ISPO), 50139 Florence, Italy; 11 Department of Clinical and Experimental Medicine, Federico II University, 80131 Naples, Italy; 12 Cancer Registry and Histopathology Unit, "Civile - M.P.Arezzo" Hospital, 97100 Ragusa, Italy; 13 HuGeF - Human Genetics Foundation - Torino, 10126 Torino, Italy; 14 Department of Epidemiology, German Institute of Human Nutrition Potsdam-Rehbrücke, 14558 Nuthetal, Germany; 15 Department of Public Health and Primary Care, University of Cambridge, Cambridge CB2 2QQ, UK; 16 Medical Research Council (MRC) Epidemiology Unit, Cambridge CB2 0QQ, UK; 17 Cancer Epidemiology Unit, Nuffield Department of Clinical Medicine, University of Oxford, Oxford OX3 7LF, UK; 18 WHO Collaborating Center for Food and Nutrition Policies, Department of Hygiene, Epidemiology and Medical Statistics, University of Athens Medical School, 11527 Athens, Greece; 19 Hellenic Health Foundation, 11527 Athens, Greece; 20 Department of Epidemiology, Harvard School of Public Health, Boston MA 02115, USA; 21 Bureau of Epidemiologic Research, Academy of Athens, 11527 Athens, Greece; 22 Julius Center, University Medical Center Utrecht, 3508 Utrecht, The Netherlands; 23 School of Public Health, Imperial College, London SW7 2AZ, UK; 24 Department of Community Medicine, University of Tromsø, 9037 Tromsø, Norway; 25 Department of Biostatistics, Faculty of Medicine, University of Oslo, 0317 Oslo, Norway; 26 Unit of Nutrition, Environment and Cancer, Cancer Epidemiology Research Program, Catalan Institute of Oncology (ICO), 08907 Barcelona, Spain; 27 Andalusian School of Public Health, 18080 Granada, Spain; 28 Consortium for Biomedical Research in Epidemiology and Public Health (CIBER Epidemiología y Salud Pública-CIBERESP), Spain; 29 Department of Epidemiology, Murcia Regional Health Council, 30008 Murcia, Spain; 30 Public Health Directorate Asturias, 33006 Oviedo, Spain; 31 Navarre Public Health Institute, 31003 Pamplona, Spain; 32 Public Health Division of Gipuzkoa, BIODonostia Research Institute, Department of Health of the Regional Government of the Basque Country, San Sebastian, Spain; 33 Department of Clinical Sciences, Lund University, 20502 Malmö, Sweden; 34 Department of Public Health and Clinical Medicine, Nutrition Research, 90185 Umeå University, Umeå, Sweden; 35 Department of Odontology, Cariology, Umeå University, 90185 Umeå, Sweden; 36 International Agency for Research on Cancer (IARC), 69008 Lyon, France; 37 Institute of Epidemiology, Helmholtz Centre Munich, 85764 Neuherberg, Germany

**Keywords:** diet, meat, mortality, cohort, Europe, cardiovascular, cancer

## Abstract

**Background:**

Recently, some US cohorts have shown a moderate association between red and processed meat consumption and mortality supporting the results of previous studies among vegetarians. The aim of this study was to examine the association of red meat, processed meat, and poultry consumption with the risk of early death in the European Prospective Investigation into Cancer and Nutrition (EPIC).

**Methods:**

Included in the analysis were 448,568 men and women without prevalent cancer, stroke, or myocardial infarction, and with complete information on diet, smoking, physical activity and body mass index, who were between 35 and 69 years old at baseline. Cox proportional hazards regression was used to examine the association of meat consumption with all-cause and cause-specific mortality.

**Results:**

As of June 2009, 26,344 deaths were observed. After multivariate adjustment, a high consumption of red meat was related to higher all-cause mortality (hazard ratio (HR) = 1.14, 95% confidence interval (CI) 1.01 to 1.28, 160+ versus 10 to 19.9 g/day), and the association was stronger for processed meat (HR = 1.44, 95% CI 1.24 to 1.66, 160+ versus 10 to 19.9 g/day). After correction for measurement error, higher all-cause mortality remained significant only for processed meat (HR = 1.18, 95% CI 1.11 to 1.25, per 50 g/d). We estimated that 3.3% (95% CI 1.5% to 5.0%) of deaths could be prevented if all participants had a processed meat consumption of less than 20 g/day. Significant associations with processed meat intake were observed for cardiovascular diseases, cancer, and 'other causes of death'. The consumption of poultry was not related to all-cause mortality.

**Conclusions:**

The results of our analysis support a moderate positive association between processed meat consumption and mortality, in particular due to cardiovascular diseases, but also to cancer.

## Background

Meat consumption has increased since World War II. While this increase has long been confined to the Western world, that is, North America, North and Western Europe, and Australia/New Zealand, meat consumption is now also on the rise in other countries, such as China, due to their economic development [1]. From a physiological perspective, a diet rich in meat has several potential nutritional benefits but also some potential adverse effects. Meat is rich in protein, iron, zinc and B-vitamins, as well as vitamin A. The bioavailability of iron and folate from meat is higher than from plant products such as grains and leafy green vegetables. The drawback, however, is the high content of cholesterol and saturated fatty acids, both of which have been shown to be positively associated with plasma low density lipoprotein (LDL) concentrations and the risk of coronary heart disease [2]. Although iron is essential for prevention of anemia, a high intake, especially of heme iron, is related to the endogenous formation of N-nitroso compounds in the gastro-intestinal tract [3,4] and, thus, may be a risk factor for some cancer entities, for example, colon cancer [5].

Some prospective studies have evaluated the association between meat intake and mortality [6-17], but several of them were studies comparing meat consumers with vegetarians [9,11,13,16]. One of the most recent studies, conducted among EPIC-Oxford participants, revealed that vegetarians as well as non-vegetarians with a health-conscious lifestyle had a statistically significantly lower mortality compared with the British general population [9]. This is similar to the results of a German cohort, in which both vegetarians and health-conscious non-vegetarians had a statistically significantly lower overall mortality compared with the general population [11]. These results indicate that the decreased mortality in vegetarians compared with the general population is in large part due to a healthy lifestyle, that is, being non-smokers, being leaner and more physically active, and so on. However, large US cohorts have reported an increased risk for early mortality among individuals with a high red and processed meat consumption compared with low meat consumption independent of smoking, obesity and other potential confounders [6,8].

Within the European Prospective Investigation into Cancer and Nutrition (EPIC) including more than 500,000 participants from ten European countries and, thus, reflecting a very heterogeneous diet, we examined the association between meat consumption and the risk for overall and cause-specific mortality.

## Methods

### Population

EPIC is a large prospective cohort study conducted in 23 centers in 10 European countries [France, Italy (Florence, Varese, Ragusa, Turin, Naples), Spain (Asturias, Granada, Murcia, Navarra, San Sebastian), The Netherlands (Bilthoven, Utrecht), United Kingdom (UK; Cambridge, Oxford), Greece, Germany (Heidelberg, Potsdam), Sweden (Malmö, Umea), Norway, and Denmark (Aarhus, Copenhagen)]. In most centers, the participants were recruited from the general population. However, the French cohort comprises female members of a health insurance program for school and university employees. Spanish and Italian participants were recruited among blood donors, members of several health insurance programs, employees of several enterprises, civil servants, but also the general population. In Utrecht and Florence, participants in mammographic screening programs were recruited for the study. In Oxford, half of the cohort consisted of 'health conscious' subjects from England, Wales, Scotland, and Northern Ireland. The cohorts of France, Norway, Utrecht, and Naples include women only [18]. Participants were recruited between 1992 and 2000 depending on the study center. At recruitment, men were 40 to 70 and women 35 to 70 years old [18]. All participants gave written informed consent to use their questionnaire data and the Internal Review Boards of the International Agency for Research on Cancer and all EPIC recruitment centers approved the analyses based on EPIC participants.

Of 511,781 apparently healthy participants at baseline, we excluded individuals with a ratio for energy intake versus energy expenditure in the top or bottom 1% (*n *= 10,197) and those with self-reported cancer, stroke or myocardial infarction at baseline (*n *= 29,300). We further excluded participants with unknown smoking status at baseline (*n *= 23,716). The analytical cohort included 448,568 participants.

### Exposure assessment

Following the results of several methodological studies conducted in the early 1990s, habitual diet over the previous twelve months was measured at recruitment by country-specific instruments designed to capture local dietary habits and to provide high compliance [18]. Seven countries adopted an extensive self-administered dietary questionnaire, which can provide data on up to 300 to 350 food items per country. In Greece, Spain and Ragusa, the dietary questionnaire was very similar in content to the above, but was administered by direct interview. A food frequency questionnaire (FFQ) and a seven-day food record were adopted in the UK. In Malmö, Sweden, a quantitative questionnaire combined with a seven-day menu book and an interview was used. Baseline food consumption, as well as ethanol and energy intake, was calculated from the dietary instruments applied in each center.

For this analysis, meats were grouped into red meat (beef, pork, mutton/lamb, horse, goat), processed meat (all meat products, including ham, bacon, sausages; small part of minced meat that has been bought as a ready-to-eat product) and white meat (poultry, including chicken, hen, turkey, duck, goose, unclassified poultry, and rabbit (domestic)). Processed meat mainly refers to processed red meat but may contain small amounts of processed white meat as well, for example, in sausages.

A set of core questions posed at recruitment that was similar in all participating centers ensured comparability of non-dietary questions and assessed information on education, medical history (including history of stroke, myocardial infarction, and cancer), alcohol consumption, physical activity, lifetime history of consumption of tobacco products including smoking status (current, past, or never smoker), type of tobacco (cigarettes, cigars, or pipe), number of cigarettes currently smoked, and age when participants started and, if applicable, quit smoking [18]. Height and weight were measured in all EPIC centers except for France, Norway, and Oxford, for which self-reported height and weight was recorded. In Oxford, self-reports were improved by using prediction equations [19].

### Outcome assessment

Information on vital status and the cause and date of death were ascertained using record linkages with cancer registries, Boards of Health, and death indices (in Denmark, Italy, the Netherlands, Norway, Spain, Sweden, and the UK) or active follow-up (in Germany, Greece, and France). Active follow-up included inquiries by mail or telephone to participants, municipal registries, regional health departments, physicians, and hospitals. Participants were censored as follows: June, 2005 (Cambridge), December 2006 (France, Varese, Turin, Naples, Granada, Murcia, Malmo, and Denmark), December 2007 (Florence, San Sebastian, Umeå and Norway), December 2008 (Ragusa, Asturias, Navarra, and the Netherlands); June 2009 (Oxford). For Germany and Greece, the end of the follow up was considered to be the last known contact or date of death, whichever came first. Cause of death was coded according to the 10^th ^Revision of the International Classification of Diseases (ICD-10). The underlying causes of death were used to estimate the cause-specific mortality: cancer (ICD-10: C00 to D48), cardiovascular diseases (I00 to I99), respiratory diseases (J30 to J98), digestive diseases (K20 to K92), and other diseases. Currently, vital status is known for 98.4% of all EPIC subjects.

### Statistical analysis

Cox proportional hazards regression was used to examine the association of meat consumption with all-cause and cause-specific mortality. To explore the shape of the risk function, we fitted a Cox proportional hazards model with restricted cubic splines for red and processed meat and poultry intake treated as continuous variables [20,21]. We specified four knot positions at 10, 20, 40, and 80 g per day of red or processed meat intake. Other knot positions were specified but did not appreciably change the curves. After examining the shape of the association between red and processed meat intake with mortality in restricted cubic spline models, we decided to choose the second category as the reference category in the categorical model (see below) for all three types of meat, that is, also for poultry for consistency reasons.

In a second step, we modeled meat intake as categorical variables as follows: red and processed meat 0 to 9.9, 10 to 19.9, 20 to 39.9, 40 t0 79.9, 80 to 159.9, and ≥160 g/day; poultry 0 to 4.9, 5 to 9.9, 10 to 19.9, 20 to 39.9, 40 to 79.9, and ≥80 g/day. Age was used as the primary time variable in the Cox models. Time at entry was age at recruitment, exit time was age when participants died, were lost to follow-up, or were censored at the end of the follow-up period, whichever came first. The analyses were stratified by sex, center, and age at recruitment in one-year categories. To adjust for lifelong tobacco smoking, we included baseline smoking status and intensity of smoking as one variable (never smokers (reference category); current cigarette smokers (three categories: 1 to 14, 15 to 24 and 25+ cigarettes/day); former smokers who stopped less than 10 years ago, 11 to 20 years ago, 20+ years ago; other smokers (one category including pipe or cigar smokers and occasional smokers)). In addition, duration of smoking in 10-year categories (≤10 (reference category), 11 to 20, 21 to 30, 31 to 40, 41 to 50, >50 years) is added as a second variable in the statistical models. We separately adjusted for the amount of smoking and the duration of smoking instead of using pack-years of smoking to differentiate better between, for example, heavy smokers of a short duration and light smokers for a long duration [22]. Additionally, all analyses were adjusted for body weight and height, energy intake, intake of alcohol (all continuous), physical activity index (active, moderately active, moderately inactive, inactive, missing) [23], and education (none or primary school completed; technical/professional school; secondary school; university degree; missing). We additionally examined the effect of mutually adjusting intake of the three types of meat for each other. We also explored meat intake in models without adjusting for total energy intake. Additionally adjusting for fruit and vegetable consumption did not appreciably change the observed associations and was not included in the main models.

In order to improve the comparability of dietary data across the participating centers, dietary intakes from the questionnaires were calibrated using a standardized 24-hour dietary recall [24,25], thus, partly correcting for over- and underestimation of dietary intakes [26]. A 24-hour dietary recall was collected from an 8% random sample of each center's participants. Dietary intakes were calibrated using a fixed effects linear model in which gender- and center-specific 24-hour dietary recall data were regressed on the questionnaire data controlling for weight, height, age, day of the week, and season of the year. The confidence intervals (CIs) of the risk estimates, obtained using calibrated data, were estimated using bootstrap sampling to take into account the uncertainty related to measurement error correction. Calibrated and uncalibrated data were used to estimate the association of meat consumption with mortality on a continuous scale.

Results of the 24-hour recalls (mean, standard error of the mean) were also used to describe the FFQ-based intake categories of red meat, processed meat, and poultry.

In our analysis, we considered cause-specific mortality in addition to overall mortality. Therefore, we fitted a competing risk model [27] which, however, resulted in similar associations as those observed in non-competing risk models for deaths from cancer, cardiovascular diseases, respiratory diseases, digestive diseases, and other diseases, and are not shown in the tables.

Results might differ between subgroups of the study population due to different health behaviors in, for example, men and women, or interactions between nutrients in different foods. Therefore, sub-analyses were performed by sex and smoking status (never, former, current), alcohol consumption (dichotomized by sex-specific median), and fruit and vegetable consumption (dichotomized by sex-specific median). Including cross-product terms along with the main effect terms in the Cox regression model tested for interaction on the multiplicative scale. The statistical significance of the cross-product terms was evaluated using the likelihood ratio test. Heterogeneity between countries was assessed using likelihood chi-square tests. We also examined whether the associations differed in the first two years and the succeeding years of follow-up.

The population attributable risk (PAR), which describes the proportion of cases that would be prevented if everyone in the study population had the reference level of the exposure, was estimated based on the formula [28]:

$$\text{PAR}=\left(\left({\sum \text{P}}_{\text{i}}\left(\text{H}{\text{R}}_{\text{i}-}1\right)\right)/\left(1+\sum {\text{P}}_{\text{i}}\left(\text{H}{\text{R}}_{\text{i}-}1\right)\right)\right)\times 100,$$

where HR_i _and P_i _are the multivariate adjusted relative risks and the prevalence, respectively, in the study population for the i^th ^exposure category (processed meat consumption 20+ g/day); I = 0: reference group (processed meat 0 to 19 g/day).

All analyses were conducted using SAS version 9.1 (SAS Institute, Cary, North Carolina).

## Results

Men and women in the top categories of red or processed meat intake in general consumed fewer fruits and vegetables than those with low intake. They were more likely to be current smokers and less likely to have a university degree (Table 1). Men with high red meat consumption consumed more alcohol than men with a low consumption, which was not seen in women. Baseline characteristics by consumption of poultry differed somewhat from the pattern observed for red and processed meat; individuals consuming more than 80 g poultry per day had a higher consumption of fruits and vegetables than those with an intake of less than 5 g per day, but there was no difference in smoking habits at baseline.

**Table 1:** Baseline information by categories of red and processed meat and poultry consumption and sex in the EPIC cohort.

	All			Red meat						Processed meat						Poultry					
				0 to 9.9 g/day			≥160 g/day			0 to 9.9 g/day			≥160 g/day			0 to 4.9 g/day			≥80 g/day		
**Men**	**Median**	**(Q1 to Q3)**		**Median**	**(Q1 to Q3)**		**Median**	**(Q1 to Q3)**		**Median**	**(Q1 to Q3)**		**Median**	**(Q1 to Q3)**		**Median**	**(Q1 to Q3)**		**Median**	**(Q1 to 3)**	
Age at recruitment (years)	52.3	45.1	-59.1	46.9	38.0	-57.0	53.3	50.1	-57.7	50.7	41.2	-60.1	49.5	43.1	-55.5	51.3	42.6	-59.6	52.3	45.9	-57.8
BMI (kg/m^2^)	26.1	24.0	-28.5	24.4	22.4	-26.6	27.0	24.9	-29.8	25.8	23.4	-28.4	27.1	24.8	-29.8	25.3	23.2	-27.7	27.4	25.1	-30.1
Energy intake (kcal/day)	2351	1947	-2816	2028	1659	-2459	3101	2680	-3616	2119	1749	-2552	3206	2739	-3750	2216	1814	-2681	2673	2240	-3162
Alcohol intake (g/day)	12.9	4.2	-29.7	8.2	1.7	-19.0	23.4	9.4	-47.2	10.2	2.3	-24.3	19.0	6.1	-40.3	10.3	2.6	-24.4	15.1	3.3	-35.0
Red meat (g/day)	51.0	26.3	-82.4	--			--			32.5	2.9	-63.8	54.0	31.7	-86.7	28.0	4.9	-59.9	65.8	34.8	-100.5
Processed meat (g/day)	33.2	14.7	-58.3	2.0	0.1	-15.9	48.2	26.8	-75.1	--			--			30.1	3.8	-59.7	29.3	13.6	-55.0
Poultry (g/day)	15.1	6.5	-27.6	0.0	0.0	-8.1	24.2	12.4	-38.4	12.2	0.0	-30.2	13.2	5.6	-26.2	--			--		
Vegetable intake (g/day)	149.6	93.0	-246.0	203.3	119.4	-306.3	198.5	130.2	-294.1	284.0	167.6	-433.5	120.4	82.3	-181.4	127.3	75.9	-216.3	233.5	155.5	-346.2
Fruit intake (g/day)	157.0	82.2	-280.7	182.0	97.8	-304.8	142.9	64.7	-254.6	251.1	139.8	-388.0	115.0	66.0	-207.6	135.9	72.0	-242.7	235.2	115.2	-391.3
	**Number**	**%**		**Number**	**%**		**Number**	**%**		**Number**	**%**		**Number**	**%**		**Number**	**%**		**Number**	**%**	
Never smoker	46191	36.3		5892	50.6		769	28.1		9602	39.6		619	28.7		10707	39.7		745	33.0	
Former smoker	47210	37.1		4161	35.7		877	32.1		8570	35.4		816	37.8		9784	36.3		909	40.2	
Current smoker	33920	26.6		1592	13.7		1090	39.8		6059	25.0		723	33.5		6449	23.9		606	26.8	
Physically inactive^a^	23258	18.3		2057	17.7		408	14.9		5751	23.7		346	16.0		4854	18.0		397	17.6	
Physically active^a^	31425	24.7		2908	25.0		856	31.3		5055	20.9		559	25.9		6295	23.4		621	27.5	
University degree	34429	27.0		5335	45.8		641	23.4		7956	32.8		522	24.2		8859	32.9		479	21.2	
**Women**	**Median**	**(Q1 to Q3)**		**Median**	**(Q1 to Q3)**		**Median**	**(Q1 to Q3)**		**Median**	**(Q1 to Q3)**		**Median**	**(Q1 to Q3)**		**Median**	**(Q1 to Q3)**		**Median**	**(Q1 to Q3)**	
Age at recruitment (years)	50.9	44.8	-57.5	47.8	38.6	-55.5	51.9	47.0	-57.2	51.1	42.0	-58.9	47.7	42.3	-53.8	50.3	42.7	-57.8	51.8	44.6	-58.0
BMI (kg/m2)	24.1	21.9	-27.2	22.9	21.0	-25.5	24.8	22.2	-28.3	23.9	21.6	-27.2	26.6	23.4	-30.7	23.3	21.2	-26.0	26.8	23.8	-30.4
Energy intake (kcal/day)	1871	1548	-2252	1718	1399	-2087	2561	2183	-3057	1730	1430	-2079	2695	2305	-3154	1789	1468	-2156	2053	1687	-2509
Alcohol intake (g/day)	3.5	0.6	-11.1	3.3	0.5	-10.3	6.1	0.8	-18.2	2.6	0.4	-10.0	4.5	0.9	-12.1	3.6	0.6	-10.9	1.9	0.0	-10.1
Red meat (g/day)	33.1	16.1	-56.7	--			--			20.4	2.2	-44.6	38.4	21.7	-65.9	13.8	1.4	-38.2	35.4	16.7	-62.8
Processed meat (g/day)	21.4	9.1	-38.5	4.6	1.0	-20.7	29.9	16.2	-51.4	--			--			15.4	2.0	-34.3	16.7	6.4	-32.4
Poultry (g/day)	12.6	4.6	-22.3	0.6	0.0	-8.1	15.1	0.0	-35.7	8.2	0.0	-19.5	13.2	5.5	-23.8	--			--		
Vegetable intake (g/day)	184.4	117.2	-284.3	219.6	133.8	-327.1	290.8	188.1	-419.2	241.9	150.7	-362.4	159.9	101.3	-244.4	179.8	108.8	-286.1	259.9	171.2	-375.6
Fruit intake (g/day)	209.9	120.1	-324.0	212.7	121.0	-331.9	226.4	122.4	-343.5	251.6	149.9	-377.7	180.5	96.3	-276.4	194.7	110.7	-308.0	257.8	150.0	-411.2
	**Number**	**%**		**Number**	**%**		**Number**	**%**		**Number**	**%**		**Number**	**%**		**Number**	**%**		**Number**	**%**	
Never smoker	186026	57.9		34149	61.7		640	58.8		53821	63.1		333	53.5		48343	58.4		1671	62.5	
Former smoker	72311	22.5		13925	25.2		219	20.1		18263	21.4		125	20.1		19377	23.4		548	20.5	
Current smoker	62910	19.6		7255	13.1		230	21.1		13271	15.5		164	26.4		15127	18.3		454	17.0	
Physically inactive^a^	69310	21.6		10273	18.6		278	25.5		22890	26.8		173	27.8		15243	18.4		838	31.4	
Physically active^a^	45458	14.2		8883	16.1		125	11.5		13497	15.8		75	12.1		13749	16.6		377	14.1	
University degree	72647	22.6		18870	34.1		295	27.1		23490	27.5		117	18.8		23999	29.0		403	15.1	

^a^as determined using the Cambridge physical activity score (including leisure time and occupational physical activity); physically inactive included the categories 'inactive' and 'moderately inactive', physically active included 'active' and 'moderately active' participants. BMI, body mass index; Q1 to Q3, range between quartiles 1 and 3.

Median follow-up time of our cohort was 12.7 years with a maximum of 17.8 years; median follow-up time was 8.5 years in cases and 12.9 years in non-cases. During the follow-up period, 26,344 study participants (11,563 men and 14,781 women) died. Of these, 5,556 died of cardiovascular diseases, 9,861 of cancer, 1,068 of respiratory diseases, 715 of digestive tract diseases, and 9,144 of other causes (including 976 who died from external causes). A high consumption of red meat was related to increased all-cause mortality (Table 2). Participants with an intake of 160+ g red meat/day had a HR = 1.37 (95% CI 1.23 to 1.54) compared with individuals with an intake of 10 to 19.9 g/day in the simple model taking into account age, study center, and sex. The association was attenuated in the multivariate model, but was still statistically significant (HR = 1.14, 95% CI 1.01 to 1.28). The association for processed meat was stronger than for red meat. In the multivariate model, the HR for high (160+ g/day) versus low intake was 1.44 (95% CI 1.24 to 1.66). Low consumption of poultry was associated with increased all-cause mortality compared with moderate consumption (Table 2), but there was no association of high poultry consumption compared with moderate consumption. Mutual adjustment for all meat groups did not appreciably change the observed associations for processed meat and poultry, but the elevated total mortality observed in the highest category of red meat consumption became statistically insignificant (Table 2). We estimated that 3.3% (95% CI 1.5 to 5.0%) of deaths could be prevented if all participants had a processed meat consumption of less than 20 g/day.

**Table 2:** Association between consumption of red and processed meat, poultry and all-cause mortality in EPIC.

Intake (g/d)	Mean (s.e.) intake^a ^(24 hour recall; g/d)		N_cases_	HR^b^	95% CI^b^	HR^c^	95% CI^c^	HR^d^	95% CI^d^
								
	Men	Women							
**Red meat**									
0 to 9.9	20.3 (2.0)	20.5 (1.0)	3175	1.05	(0.99, 1.10)	1.07	(1.01, 1.13)	1.04	(0.99, 1.10)
10 to 19.9	35.5 (2.0)	25.9 (0.9)	2774	1.00	(ref)	1.00	(ref)	1.00	(ref)
20 to 39.9	47.9 (1.5)	33.1 (0.7)	6459	1.02	(0.98, 1.07)	1.01	(0.97, 1.06)	1.01	(0.97, 1.06)
40 to 79.9	62.3 (1.4)	44.8 (0.8)	8935	1.04	(0.99, 1.09)	0.99	(0.94, 1.03)	0.99	(0.94, 1.03)
80 to 159.9	81.0 (2.0)	55.9 (1.5)	4639	1.15	(1.09, 1.21)	1.03	(0.98, 1.09)	1.03	(0.97, 1.08)
160+	110.8 (7.7)	70.9 (10.8)	362	1.37	(1.23, 1.54)	1.14	(1.01, 1.28)	1.10	(0.98, 1.24)
**Processed meat**									
0 to 9.9	14.9 (0.9)	14.3 (0.5)	6236	1.00	(0.96, 1.04)	1.04	(0.99, 1.08)	1.01	(0.97, 1.06)
10 to 19.9	37.5 (1.5)	26.9 (0.6)	4683	1.00	(ref)	1.00	(ref)	1.00	(ref)
20 to 39.9	51.1 (1.2)	36.1 (0.6)	7301	1.06	(1.03, 1.11)	1.03	(0.99, 1.07)	1.03	(0.99, 1.07)
40 to 79.9	71.6 (1.4)	46.6 (0.9)	5997	1.17	(1.12, 1.22)	1.09	(1.05, 1.14)	1.09	(1.04, 1.13)
80 to 159.9	90.7 (2.4)	57.8 (2.5)	1904	1.36	(1.28, 1.44)	1.21	(1.14, 1.28)	1.20	(1.13, 1.28)
160+	121.3 (7.7)	71.0 (12.2)	223	1.74	(1.51, 2.00)	1.44	(1.24, 1.66)	1.43	(1.24, 1.64)
**Poultry**									
0 to 4.9	9.7 (0.8)	10.5 (0.5)	6973	1.08	(1.04, 1.13)	1.08	(1.04, 1.13)	1.08	(1.03, 1.12)
5 to 9.9	11.4 (1.0)	12.5 (0.6)	4568	1.00	(ref)	1.00	(ref)	1.00	(ref)
10 to 19.9	20.4 (1.1)	16.0 (0.6)	7211	0.97	(0.94, 1.01)	0.98	(0.95, 1.02)	0.98	(0.95, 1.02)
20 to 39.9	22.4 (1.1)	22.4 (0.8)	4563	0.95	(0.91, 0.99)	0.97	(0.93, 1.02)	0.97	(0.93, 1.01)
40 to 79.9	36.6 (2.2)	26.3 (1.4)	2702	0.95	(0.90, 1.00)	0.97	(0.93, 1.03)	0.97	(0.92, 1.02)
80+	50.3 (6.1)	35.6 (6.2)	327	1.03	(0.92, 1.15)	1.05	(0.94, 1.18)	1.05	(0.94, 1.18)

**^a^**means (and SD) computed from 24-hour recalls based on categories from FFQs; ^b^stratified by age (one-year age groups), sex, study center; **^c^**stratified by age (one-year age groups), sex, study center, adjusted for education (five categories), body weight (continuous), body height (continuous), total energy intake (continuous), alcohol consumption (continuous), physical activity (four categories), smoking status (seven categories), smoking duration (six categories); **^d^**stratified by age (one-year age groups), sex, study center, adjusted for education (five categories), body weight (continuous), body height (continuous), total energy intake (continuous), alcohol consumption (continuous), physical activity (four categories), smoking status (seven categories), smoking duration (six categories), meat intake mutually adjusted for each other. CI, confidence interval; FFQs, food frequency questionnaires; HR, hazard rate; N, number; s.e., standard error.

**Figure 1:**
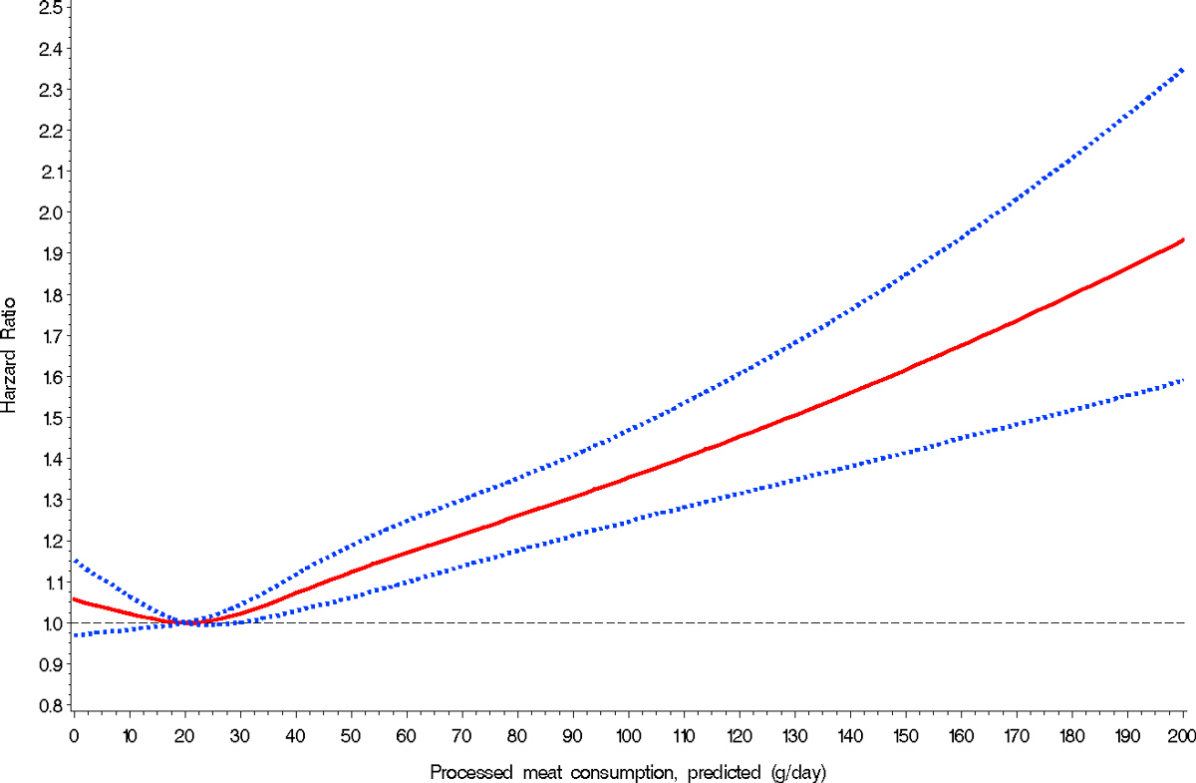
**Nonparametric regression curve for the relation of processed meat intake at recruitment with all-cause mortality, European Prospective Investigation into Cancer and Nutrition (EPIC), 1992-2009**. Solid line, effect estimate; dotted lines, 95 percent confidence interval.

We also evaluated the association between meat consumption and all-cause mortality in two continuous models, that is, obtaining uncalibrated and calibrated risk estimates (Table 3). Similarly to the observation of no association between red meat consumption and all-cause mortality in the multivariable categorical model, we observed no statistically significant association in the continuous models either. While the associations were similar in the uncalibrated and calibrated models for red meat and poultry, the association between processed meat consumption and all-cause mortality was stronger in the calibrated model: per 50 g increase in daily processed meat consumption, the HR for all-cause mortality was 1.18 (95% CI 1.11 to 1.25). In the calibrated spline models, we observed significantly higher all-cause mortality with higher consumption of processed meat and no statistically significant association with red meat or poultry intake (Figure 1). However, all-cause mortality was higher among participants with very low or no red meat consumption.

**Table 3:** Association between consumption of red and processed meat, and poultry and all-cause mortality in EPIC.

	Observed	Calibrated
	HR^a ^(95% CI)	HR^a ^(95% CI)
Red meat (per 100 g)	1.02 (0.98 to 1.06)	1.02 (0.98 to 1.06)
Processed meat (per 50 g)	1.09 (1.06 to 1.12)	1.18 (1.11 to 1.25)
Poultry (per 50 g)	0.96 (0.92 to 0.99)	0.95 (0.87 to 1.04)

**^a^**stratified by age (one-year age groups), sex, study center, adjusted for education (five categories), body weight (continuous), body height (continuous), total energy intake (continuous), alcohol consumption (continuous), physical activity (four categories), smoking status (seven categories), smoking duration (six categories); CI, confidence interval; HR, hazard rate.

We also explored the association of meat intake with mortality in models without adjusting for total energy intake. However, the results were identical for models not including (data not shown) and including total energy intake. Results were also similar for models including total energy and fruit and vegetable intake. The associations between red or processed meat or poultry intake and all-cause mortality were also similar for the first two years or after the first two years of follow-up (data not shown).

For processed meat, for which we observed statistically significant associations with overall mortality, we examined whether this effect differed by sub-groups of our population. We did not observe statistically significant effect modification by sex (Table 4), with similarly increased all-cause mortality in both sexes, although the association was statistically significant only among men (HR = 1.35, 95% CI 1.16 to 1.58, 160+ versus 10 to 19.9 g/day), but not among women (HR = 1.38, 95% CI 0.95 to 2.00; *P*-interaction 0.88). This may be due to the relatively small number of deaths among women in the highest processed meat consumption category (29 women; 194 men). There was also a statistically significant interaction between smoking and processed meat consumption (*P*-interaction 0.01), with mortality being significantly increased among former (HR = 1.68, 95% CI 1.29 to 2.18) and current smokers (HR = 1.47, 95% CI 1.18 to 1.83), but there was no association among never smokers (HR = 1.24, 95% CI 0.89 to 1.72). However, the small number of deaths among never smokers has to be taken into account (*n *= 44; 72 among former and 107 among current smokers in the top consumption category). We observed a statistically significant interaction with body mass index, such that the association between processed meat consumption and all-cause mortality was stronger in lean than in overweight and obese participants (*P*-interaction 0.04). Those with a lower fruit and vegetable intake (below median intake) had a higher overall mortality in the highest consumption category of processed meat (160+ g/day) as compared to subjects with a fruit and vegetable intake above the median intake (*P*-interaction 0.001).

**Table 4:** Association between processed meat consumption and all-cause mortality by sex, alcohol consumption, BMI, smoking status, and fruit and vegetable consumption.

		Intake (g/day)						
			
		0-9.9	10-19.9	20-39.9	40-79.9	80-159.9	160+	*P-interaction*
Sex								
Males	HR	0.99	1.00	1.00	1.04	1.13	1.32	
	(95% CI)	(0.91, 1.07)	(ref.)	(0.94, 1.07)	(0.98, 1.12)	(1.04, 1.22)	(1.12, 1.54)	
Females	HR	1.05	1.00	1.04	1.09	1.22	1.37	
	(95% CI)	(1.00, 1.10)	(ref.)	(0.99, 1.09)	(1.04, 1.16)	(1.10, 1.34)	(0.94, 2.00)	*0.88*
Alcohol^a^								
<median	HR	1.00	1.00	1.02	1.08	1.19	1.35	
	(95% CI)	(0.94 to 1.06)	(ref.)	(0.97 to 1.08)	(1.02 to 1.15)	(1.09 to 1.30)	(1.09 to 1.69)	
≥median	HR	1.03	1.00	1.05	1.1	1.19	1.45	*0.71*
	(95% CI)	(0.97 to 1.10)	(ref.)	(0.99 to 1.11)	(1.03 to 1.17)	(1.09 to 1.31)	(1.19 to 1.77)	
BMI								
<25 kg/m^2^	HR	1.06	1.00	1.03	1.10	1.15	1.63	
	(95% CI)	(1.00 to 1.13)	(ref.)	(0.97 to 1.09)	(1.03 to 1.18)	(1.04 to 1.28)	(1.27 to 2.09)	
≥25 kg/m^2^	HR	0.99	1.00	1.04	1.09	1.25	1.38	
	(95% CI)	(0.93 to 1.05)	(ref.)	(0.99 to 1.10)	(1.03 to 1.15)	(1.15 to 1.35)	(1.15 to 1.65)	*0.04*
Smoking status								
Never	HR	1.02	1.00	0.99	1.01	1.15	1.24	
	(95% CI)	(0.96, 1.09)	(ref.)	(0.93, 1.05)	(0.94, 1.08)	(1.03, 1.29)	(0.89, 1.72)	
Former	HR	1.03	1.00	1.07	1.14	1.26	1.68	
	(95% CI)	(0.95, 1.11)	(ref.)	(0.99, 1.14)	(1.06, 1.24)	(1.12, 1.41)	(1.29, 2.18)	
Current	HR	1.04	1.00	1.08	1.15	1.26	1.47	
	(95% CI)	(0.95, 1.13)	(ref.)	(1.01, 1.16)	(1.07, 1.25)	(1.13, 1.40)	(1.18, 1.83)	*0.01*
Fruits and vegetables								
<median	HR	1.1	1.00	1.04	1.1	1.2	1.53	
	(95% CI)	(1.04 to 1.17)	(ref.)	(0.99 to 1.10)	(1.04 to 1.16)	(1.11 to 1.30)	(1.29 to 1.83)	
≥median	HR	0.99	1.00	1.03	1.08	1.2	1.27	
	(95% CI)	(0.94 to 1.05)	(ref.)	(0.97 to 1.08)	(1.01 to 1.15)	(1.09 to 1.32)	(0.99 to 1.63)	*0.001*

^a^Cut-offs: alcohol: 14.8 g (men), 3.8 g (women); fruits and vegetables: 326 g (men), 414 g (women). BMI, body mass index; CI, confidence interval; HR, hazard rate.

No statistically significant heterogeneity between countries was observed for the associations of processed meat and poultry consumption with mortality (*P*-values >0.05), but was observed for the association of red meat intake with mortality (*P*-value 0.006). This heterogeneity was not driven by risk estimates from a particular country (data not shown).

Very high consumption of red meat was associated with a non-significantly increased cancer mortality, but not with deaths due to cardiovascular diseases, respiratory diseases, diseases of the digestive tract, or any other cause of deaths (Table 5). However, the increase in risk was not observed in the continuous model. In contrast, participants who consumed 160+ g of processed meat per day had an increased risk of dying of cardiovascular diseases compared with those who consumed only moderate amounts (10 to 19.9 g/day; HR = 1.72, 95% CI 1.29 to 2.30); this association was also observed in the continuous model, even after correcting for measurement error (HR = 1.30, 95% CI 1.17 to 1.45 per 50 g/day). There was also a significant positive association between processed meat consumption and risk of dying from cancer (HR = 1.11, 95% CI 1.03 to 1.21 per 50 g/day) or other causes of death (HR = 1.22, 95% CI 1.11 to 1.34 per 50 g/day). Since the results of the categorical model deviated from the continuous model, we reexamined the association between processed meat intake and cancer risk using the lowest consumption category (0 to 9.9 g/d) as reference and observed a statistically significantly increased risk for cancer mortality for those who consumed 80 to 159.9 g/day (HR = 1.12, 95% CI 1.01 to 1.24) and a non-significantly increased risk in the highest consumption category (HR = 1.19, 95% CI 0.93 to 1.51). Although for some categories of processed meat intake a positive association with mortality from respiratory diseases and digestive tract diseases was obtained, the continuous models failed to reach statistical significance. Poultry consumption was not consistently associated with deaths from any of these causes.

**Table 5:** Association between meat consumption and cause-specific mortality in EPIC.

	Cardiovascular diseases			Cancer			Respiratory diseases			Digestive tract			Other cause of death		
Intake (g/d)	N_cases_	HR^a^	95% CI	N_cases_	HR^a^	95% CI	N_cases_	HR^a^	95% CI	N_cases_	HR^a^	95% CI	N_cases_	HR^a^	95% CI
Red meat															
0 to 9.9	695	1.03	(0.92, 1.16)	1077	1.04	(0.95, 1.14)	164	1.09	(0.84, 1.41)	74	0.81	(0.58, 1.14)	1019	1.10	(1.00, 1.21)
10 to 19.9	613	1.00	(ref.)	1052	1.00	(ref.)	116	1.00	(ref.)	74	1.00	(ref.)	833	1.00	(ref.)
20 to 39.9	1395	1.01	(0.91, 1.11)	2477	1.04	(0.96, 1.12)	227	0.86	(0.68, 1.09)	170	0.95	(0.72, 1.25)	1971	1.02	(0.94, 1.11)
40 to 9.9	1862	1.02	(0.92, 1.12)	3353	0.99	(0.91, 1.06)	357	0.92	(0.73, 1.16)	239	0.84	(0.64, 1.11)	2800	0.98	(0.90, 1.07)
80 to 159.9	924	1.04	(0.93, 1.17)	1759	1.03	(0.95, 1.13)	194	1.05	(0.81, 1.37)	144	0.88	(0.64, 1.21)	1433	1.03	(0.94, 1.14)
160+	67	1.07	(0.82, 1.40)	143	1.21	(1.00, 1.46)	10	0.80	(0.40, 1.60)	14	1.02	(0.55, 1.90)	112	1.17	(0.95, 1.44)
Per 100 g/day															
Observed		1.10	(1.00, 1.19)		1.01	(0.94, 1.08)		1.02	(0.83, 1.24)		1.04	(0.82, 1.32)		1.01	(0.95, 1.09)
Calibrated		1.09	(1.00, 1.18)		1.00	(0.94, 1.07)		1.06	(0.88, 1.28)		1.03	(0.83, 1.29)		1.02	(0.94, 1.10)
															
Processed meat															
0 to 9.9	1635	1.06	(0.96, 1.16)	2223	0.96	(0.90, 1.03)	322	1.11	(0.90, 1.37)	161	1.08	(0.83, 1.41)	1654	1.10	(1.02, 1.18)
10 to 19.9	855	1.00	(ref.)	1848	1.00	(ref.)	171	1.00	(ref.)	112	1.00	(ref.)	1547	1.00	(ref.)
20 to 39.9	1335	1.05	(0.96, 1.15)	2745	1.01	(0.95, 1.07)	287	1.21	(0.99, 1.47)	209	1.30	(1.02, 1.64)	2496	1.03	(0.97, 1.10)
40 to 79.9	1222	1.16	(1.05, 1.28)	2252	1.03	(0.96, 1.10)	220	1.29	(1.04, 1.61)	178	1.37	(1.06, 1.78)	1889	1.09	(1.02, 1.18)
80 to 159.9	453	1.35	(1.18, 1.54)	714	1.08	(0.98, 1.19)	60	1.27	(0.91, 1.77)	48	1.18	(0.80, 1.73)	526	1.28	(1.14, 1.43)
160+	56	1.72	(1.29, 2.30)	79	1.15	(0.90, 1.46)	8	1.73	(0.82, 3.65)	7	1.58	(0.70, 3.54)	56	1.64	(1.24, 2.18)
Per 50 g/day															
Observed		1.15	(1.09, 1.21)		1.06	(1.02, 1.10)		1.10	(0.98, 1.25)		1.04	(0.91, 1.20)		1.11	(1.06, 1.15)
Calibrated		1.30	(1.17, 1.45)		1.11	(1.03, 1.21)		1.22	(0.97, 1.54)		1.09	(0.82, 1.47)		1.22	(1.11, 1.34)
															
Poultry															
0 to 4.9	1494	1.05	(0.96, 1.15)	2502	1.10	(1.03, 1.17)	297	1.11	(0.91, 1.35)	189	1.03	(0.81, 1.30)	2220	1.09	(1.01, 1.17)
5 to 9.9	982	1.00	(ref.)	1706	1.00	(ref.)	184	1.00	(ref.)	132	1.00	(ref.)	1383	1.00	(ref.)
10 to 19.9	1565	1.00	(0.92, 1.09)	2649	1.00	(0.94, 1.06)	294	0.99	(0.82, 1.20)	191	0.89	(0.71, 1.12)	2286	0.98	(0.91, 1.05)
20 to 39.9	907	0.92	(0.83, 1.01)	1853	0.99	(0.93, 1.07)	153	0.95	(0.75, 1.20)	121	0.85	(0.66, 1.11)	1344	1.02	(0.95, 1.11)
40 to 79.9	541	0.90	(0.81, 1.01)	1024	1.01	(0.93, 1.10)	123	1.05	(0.82, 1.34)	68	0.85	(0.63, 1.16)	853	1.00	(0.92, 1.10)
80+	67	0.94	(0.73, 1.21)	127	1.00	(0.83, 1.20)	17	1.37	(0.82, 2.28)	14	1.42	(0.80, 2.50)	82	1.10	(0.88, 1.39)
Per 50 g/day															
Observed		0.93	(0.85, 1.01)		0.97	(0.91, 1.03)		1.21	(1.06, 1.38)		0.89	(0.71, 1.13)		1.01	(0.95, 1.07)
Calibrated		0.84	(0.69, 1.03)		0.98	(0.82, 1.16)		1.32	(1.02, 1.73)		0.72	(0.41, 1.29)		1.05	(0.91, 1.21)

**^a^**stratified by age (one-year age groups), sex, study center, adjusted for education (five categories), body weight (continuous), body height (continuous), total energy intake (continuous), alcohol consumption (continuous), physical activity (four categories), smoking status (seven categories), smoking duration (six categories); CI, confidence interval; HR, hazard rate; N, number.

## Discussion

In the EPIC cohort, a high consumption of processed meat was related to moderately higher all-cause mortality. After correction for measurement error, red meat intake was no longer associated with mortality, and there was no association with the consumption of poultry. Processed meat consumption was associated with increased risk of death from cardiovascular diseases and cancer.

The largest study so far, the National Institutes of Health-American Association of Retired Persons (NIH-AARP) cohort in the US, reported positive associations of both red and processed meat consumption with risk for all-cause mortality [8]. In that cohort, the association was stronger for red meat than for processed meat intake, which might be due to the fact that red meat in that US cohort also included processed meat. Similarly, in the Nurses' Health Study and the Health Professionals Follow-up Study (HPFS), high red meat intake was related to higher all-cause mortality [6]. The effect was similar for unprocessed and processed red meat. Similar associations were reported in other [10,14,17], but not all, studies [15]. Also, several vegetarian studies did not find increased all-cause mortality among non-vegetarians compared with vegetarians [9,11,13,16]. The EPIC results do not show the lowest relative risks (RRs) for subjects in the lowest meat intake category, but a slight J-shaped association with the lowest risk among subjects with low-to-moderate meat consumption. This was observed for red meat and poultry. Also, taking into account the results from the studies that evaluated vegetarian and low-meat diets, it appears that a low - but not a zero - consumption of meat might be beneficial for health. This is understandable as meat is an important source of nutrients, such as protein, iron, zinc, several B-vitamins as well as vitamin A and essential fatty acids (linoleic acid and to a minor extent eicosapentaenoic and docosahexaenoic acids also). A sub-optimal supply of some of these nutrients due to an unbalanced type of vegetarian diet seems possible and might be associated with an increased risk for morbidity and mortality. However, support for this hypothesis from the literature is not strong, especially when looking at the population level. Alternatively, subjects with very moderate meat consumption may be the group with the highest proportion of health-conscious subjects who also try to optimize their diet (as part of a healthy lifestyle).

In contrast to the US results, we observed a consistent association between processed meat consumption and total mortality but not between red meat consumption and total mortality. Processed meats such as sausages, salami and bacon have a higher content of saturated fatty acids and cholesterol than fresh red meat; the latter is often consumed after removing the visible fat tissue, whereas the proportion of fat in sausages often reaches 50% of the weight or even more. Both high saturated fat and cholesterol intake have been found to be related to the risk of coronary heart disease [2]. Also, processed meat is treated by salting, curing, or smoking in order to improve the durability of the food and/or to improve color and taste. These processes, however, lead to an increased intake of carcinogens or their precursors (polycyclic aromatic hydrocarbons, heterocyclic aromatic amines, nitrosamines) or to a high intake of specific compounds possibly enhancing the development of carcinogenic processes (for example, nitrite).

We estimated that 3.3% of all deaths could be prevented if processed meat consumption were below 20 g/day. In the AARP cohort, the preventable fraction was estimated to be much higher, that is, 20% if women decreased their processed meat consumption to less than 1.6 g/1,000 kcal/day (the authors did not state the preventable fraction for men [8]). The preventable fraction was estimated to be 9.3% in the HPFS and 7.6% in the Nurses' Health Study if the participants lowered their red meat (processed and unprocessed) consumption to less than 0.5 servings per day. The difference between the US studies and our result is likely due to the stronger risk estimates observed in the US cohorts compared with our cohort, but may also be explained by higher meat consumption in the US than in Europe.

As in the US cohorts, EPIC participants with a high processed meat intake had an increased risk of cardiovascular and cancer mortality. We have previously reported an increased risk of colorectal [29] and gastric [30] cancer with high meat, in particular processed meat, consumption. However, in contrast to the US cohorts [6,8], there was no statistically significant association of red meat consumption with risk of cancer or cardiovascular mortality. Also, in the Japan Collaborative Cohort Study, meat consumption up to 100 g/day was not related to increased mortality from cardiovascular disease [7].

The EPIC study has several strengths including its prospective design, the large sample size and the assessment of diet using two different methods, that is, dietary questionnaires and a 24-hour dietary recall in a representative sub-sample of the cohort. In a series of validation studies, correlation coefficients for meat intake between 12 24-hour recalls and food questionnaires ranged between 0.4 and 0.7 [31]. The single 24-hour recalls in a representative sample of the cohorts allow for partly correcting for systematic over- and underestimation of dietary intakes [32,33]. It is, therefore, important to note that the impact of the calibration method in our study was such that the risk estimates from the calibrated data are usually stronger than the non-calibrated results. Nevertheless, measurement error may still have an effect on calibrated RRs to a certain extent because the error structure in the reference method is not entirely independent of that in the FFQ [34,35]. A further methodological strength of the EPIC cohort is the inclusion of individuals from 10 European countries with distinctly diverging meat consumption habits [36]. A high between-person variation in diet decreases the impact of measurement error and enables the detection of only modest diet-disease relationships. We did explore meat intake in models with and without adjusting for total energy intake. In models adjusting for energy intake, meat intake must substitute the intake of other non-specified energy-providing foods. However, the results were identical for models including and not including total energy intake and also for models including total energy and fruit and vegetable intake, which have also been considered important in the development of chronic diseases. The results observed in this study were, thus, robust in a number of different models with different interpretation. Lastly, loss to follow-up is negligible as vital status is known for 98% of the cohort.

We cannot exclude residual confounding, in particular due to incomplete adjustment for active and passive smoking. The sub-group analysis for processed meat showed heterogeneity according to smoking, with significant associations only in former and current smokers and no significant associations in never smokers, which is compatible with residual confounding by smoking. Although EPIC includes ten European countries with a wide range of dietary behaviors, we observed relatively little heterogeneity in the association between meat consumption and overall mortality.

We relied on mortality information from death certificates but cause of death as coded on death certificates is not perfect. Deaths due to cancer are most correctly diagnosed, whereas deaths due to coronary heart disease tend to be overrepresented and respiratory diseases might be underrepresented [37-39].

## Conclusions

The results of our analyses suggest that men and women with a high consumption of processed meat are at increased risk of early death, in particular due to cardiovascular diseases but also to cancer. In this population, reduction of processed meat consumption to less than 20 g/day would prevent more than 3% of all deaths. As processed meat consumption is a modifiable risk factor, health promotion activities should include specific advice on lowering processed meat consumption.

## Abbreviations

AARP: American Association of Retired Persons; CI: confidence interval; EPIC: European Prospective Investigation into Cancer and Nutrition; FFQ: food frequency questionnaire; HPFS: Health Professionals Follow-up Study; HR: hazard rate; ICD-10: 10^th ^Revision of the International Classification of Diseases; LDL: low density lipoprotein; NIH: National Institutes of Health; PAR: population attributable risk; RR: relative risk.

## Competing interests

The authors declare that they have no competing interests.

## Authors' contributions

ER is the overall coordinator of the EPIC study, which he conceptualized, designed, and implemented in collaboration with the main investigators in the collaborating centers: Denmark: ATjønneland, KO; France: MCBR, FCC; Germany: RK, HB; Greece: ATrichopoulou, DT; Italy: VK, DP, SP, RT; *Netherlands: *HBBdM, PHMP; Spain: MJS, AB; Sweden: GH; UK: KTK, NW, TJK; IARC: IR. All authors contributed to recruitment, data collection/acquisition and/or biological sample collection, and are responsible for the ongoing follow-up and management of the EPIC cohort. This analysis was initiated and supervised by JL. The article was written by SR with assistance from JL, KO, and HBBdM, taking into account the comments and suggestions of the coauthors. All coauthors had the opportunity to comment on the analysis and interpretation of the findings and approved the final version for publication.

## Pre-publication history

The pre-publication history for this paper can be accessed here:

http://www.biomedcentral.com/1741-7015/11/63/prepub
